# Supplementary material for: Sialic Acids on Tumor Cells Modulate IgA Therapy by Neutrophils via Inhibitory Receptors Siglec-7 and Siglec-9
Source: Cancers (Basel). 2023 Jun 29;15(13):3405. doi: 10.3390/cancers15133405 (PMC10341145; doi:10.3390/cancers15133405)
Supplement: Supplementary file 1 [file cancers-15-03405-s001.zip › cancers-2460208-supplementary.pdf]

Supplementary Figure 1

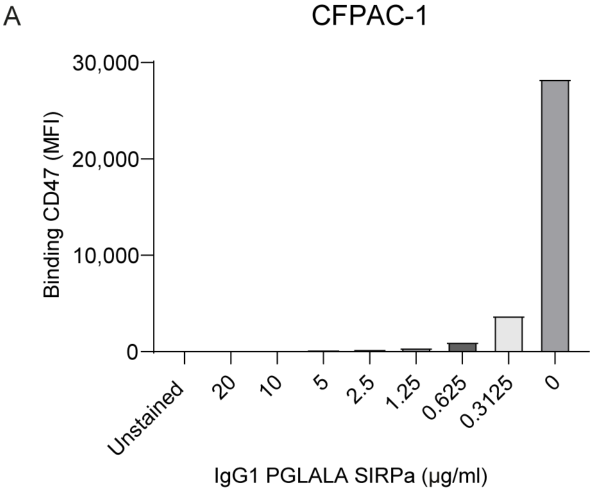

Supplementary Figure S1. CD47 binding competition assay with CFPAC-1 (CD47 high) cells. Cells were pre-incubated with titrated concentrations of SIRPα fusion protein at RT for 30 minutes. Subsequently, a PE-labeled CD47 antibody (CC2C6), targeting the same binding epitope as SIRPα, was introduced to compete with the SIRPα fusion protein. The displacement of SIRPα fusion protein was measured using flow cytometry after a 45-minute incubation period.

Supplementary Figure 2

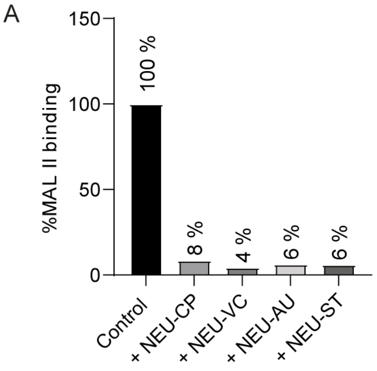

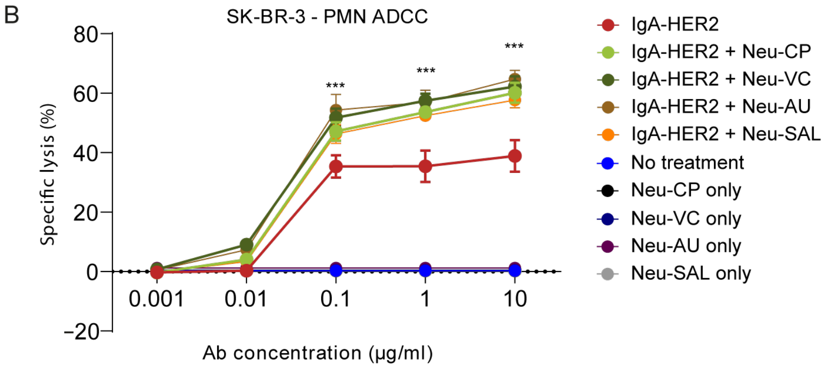

Supplementary Figure S2. Neuraminidase treatment reduced surface sialic acids and improved ADCC.

SK-BR-3 cells were pretreated with 0.1 U/ml neuraminidase (NEU-CP, NEU-VC, NEU-AU, NEU-ST) for 1 hour in a humidified shaking incubator at 125 rpm, 37°C and 8% CO_2. **(A)** Expression of α2,3-linked sialic acids was determined using MAL II lectin binding by flow cytometry and shown in percentage of MAL II binding. **(B)** PMN-mediated ADCC against SK-BR-3 cells by IgA HER2 (trastuzumab) in concentrations ranging from 0 µg/ml to 10 µg/ml. Specific lysis was determined after 4 hours in a ^{51}Cr release assay. PMNs were co-cultured with the tumor cells at an E:T ratio of 40:1. The mean ± SD specific lysis of a technical triplicate of single donor is shown. At least n=3 independent experiments are represented by one representative graph. ns>0.05, ***p < 0.001, by two-way ANOVA followed by followed by Tukey's post-hoc test.

Supplementary figure 3

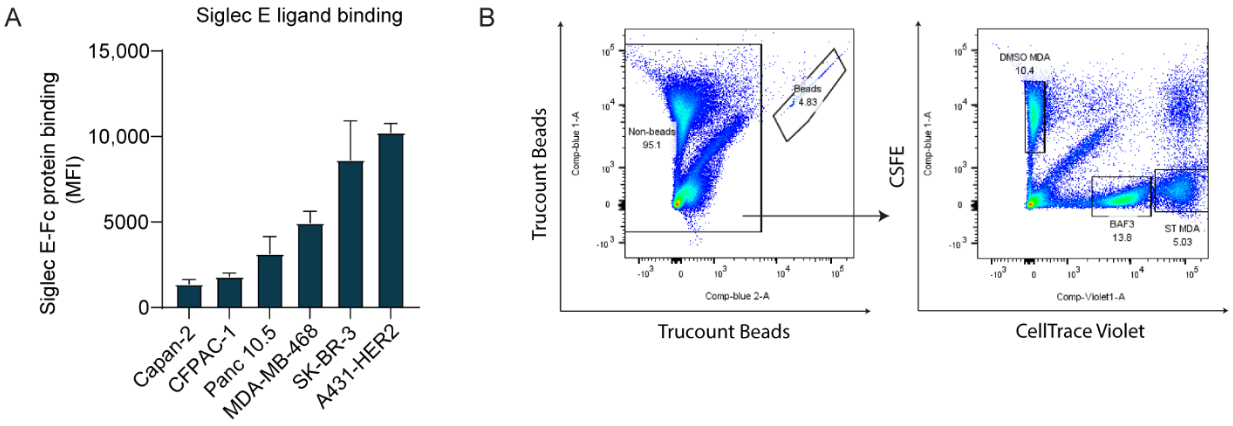

Supplementary Figure S3. MDA-MB-468 short i.p. xenograft model (A) Detection of Siglec-E ligands on a panel of tumor cells using 10 µg/ml Siglec-E-Fc protein by flow cytometry. **(B)** Gating strategy of cells recovered from the peritoneum are quantified using Trucount tubes. The cells are first separated from the Blue-1/Blue-2 double positive beads. DMSO treated MDA-MB-468 cells are CSFE+, while Ba/F3 target negative cells are CTV low, and STinh treated MDA-MB-468 cells are CTV high.
